# From headache to kidney tumor; an example of von Hippel- Lindau disease

**DOI:** 10.12861/jrip.2015.21

**Published:** 2015-09-01

**Authors:** Mohammed Asserraji, Abdennasser El Kharras

**Affiliations:** ^1^Dialysis Unit, First Medico-Surgical Hospital, Agadir, Morocco; ^2^Department of Radiology, First Medico-Surgical Hospital, Agadir, Morocco

**Keywords:** Von Hippel-Lindau disease, Central nervous system neoplasms, Renal cell carcinoma

## Abstract

**Background:** von Hippel-Lindau disease (VHL) is a rare genetic condition caused by the mutation of the VHL tumor suppressor gene and predisposing to various benign or malignant tumors involving mainly central nervous system (CNS) and retinal hemangioblastomas (RHB). Although considered as occult, multiple renal cysts and renal cell carcinoma (RCC) are frequent in VHL, occurring in nearly two-thirds of patients. RCC is the major neoplasm and the main cause of death in patients with this condition.

**Case:** In this report, we present a case of an occult kidney tumor revealed by neurological symptoms of cerebellar hemangioblastoma.

**Conclusion:** Kidney tumor was diagnosed incidentally on abdominal tomography and confirmed by histopathology analysis.

**Implication for health policy/practice/research/medical education:**

von Hippel-Lindau disease (VHL) is a rare hereditary disorder with multiorgan involvement and variable expression. This condition could involve the renal tissue and may be difficult to recognize at the right time.

## Introduction

von Hippel-Lindau disease (VHL) is a hereditary disease with multiorgan involvement and variable expression (1). The disease predisposes to various benign or malignant tumors: central nervous system (CNS), renal cysts and renal cell carcinoma (RCC), retinal hemangioblastomas (RHB), and also pancreatic tumors and cysts (1,2).

In this report, we present a case of VHL disease involving first CNS with symptoms of intracranial hypertension. Kidney tumor was diagnosed incidentally on abdominal tomography and confirmed by histopathology analysis.

## Case presentation

A 28-year-old woman with intense headache presented to our hospital to undergo a brain scan. Family history revealed that her father had died from cerebral vascular accident. General physical examination showed a very thin female with a body mass index of 21 kg/m^2^ and blood pressure of 122/72 mm Hg and signs of intracranial hypertension (headache, vomiting, and photophobia). There was no evidence of any neurological deficit. The abdominal examination showed epigastric mass and the renal angles were full with palpable kidneys. Ocular fundi were normal. Other aspects of the clinical examination were normal.

**Figure 1:**
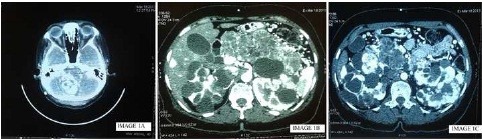
Computerized tomography (CT) scan of the brain: cerebellum hemangioblastoma (A) and the abdomen: pancreas cystic, multiple renal cysts, (B) and suspicious renal carcinoma (C).

**Figure 2:**
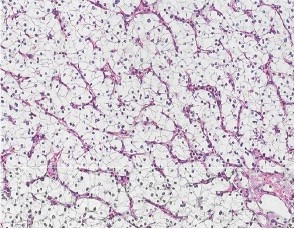
Histopathology analysis showing tumoral proliferation of clear cells with a vegetable cell-like appearance and highly vascular stroma (HE ×200).

Laboratory parameters showed normal liver and kidney functions. Computerized tomography (CT) scan of the brain showed a suspect image suggestive of cerebellar hemangioblastoma (Figure 1A). Additional CT scan of the abdomen revealed cystic lesions in the head and body of the pancreas, bilateral multiple simple and complex renal cysts, (Figure 1B) and suspicious renal carcinoma on later images (Figure 1C). Symptomatic treatment was instituted waiting for complete surgical resection of the cerebellar tumor. Nephrectomy was also performed. The histopathological findings confirmed the presence of RCC of the clear cell type (Figure 2).

## Discussion

Dr. Eugene von Hippel, a German ophthalmologist and Dr. Avrid Lindau a Swedish pathologist (3), were the first to describe VHL at the beginning of the 20th century. VHL is an autosomal dominant syndrome associated with a variety of tumors including retinal angioma, CNS hemangioblastomas, pheochromocytomas, RCCs, and ear, nose and throat (ENT) tumors with very high phenotypic variability and age-dependent penetrance (4). Early diagnosis of this condition is important for adequate screening and follow‑up (5).

VHL results primarily from a mutation of a tumor suppressor gene located on chromosome 3p25-26 (identified in 1993). This mutation could be familial (80%) or sporadic in 20% of patients (de novo mutation). The protein produced by this gene is involved in tumor suppression by degradation of hypoxia inducible factor proteins (HIF). The HIF proteins promote pathological tumor growth via up-regulation of glucose metabolism, angiogenesis and mitogenesis (6). This disease affects 1 in 36000 newborns worldwide (7).

With a confirmed familial history of VHL, the diagnosis of VHL could be made by finding a single VHL tumor (retinal or CNS hemangioblastoma, clear cell RCC, pheochromocytoma, pancreatic endocrine tumor or endolymphatic sac tumor). Without a positive familial history, VHL is considered as a sporadic condition and all of the tumors typically found in familial VHL can occur as a sporadic event. Clinical diagnosis requires the presence of two tumors (two hemangioblastomas or a hemangioblastoma and a visceral tumor) (7).

## Conclusion

We report in this paper an occult kidney tumor revealed by neurological symptoms of cerebellar hemangioblastoma. VHL is the leading cause of inherited kidney cancer. Majority of VHL cases reveal renal pathology on detailed evaluation, showing the significant burden of renal illness in these patients. Patients with neurological complications must be referred to an urologist/nephrologist for review and renal diagnostic imaging. Standardized screening program of patients with VHL and their family with genetic confirmation of VHL may be necessary.

RCC in VHL can occur alone or combined with complex cystic masses. The histopathology examination of nephrectomy showed clear cell carcinoma that was the exclusively demonstrated VHL-associated renal tumor (Figure 2). RCC occurring in VHL is known to have similar growth kinetics as those of sporadic one (1). This highlights the importance of renal screening in patients with VHLD. In conclusion, RCC is the major cause of death in patients with VHLD. VHL patients must undergo renal disease screening.

## Authors’ contribution

All authors wrote the paper equally.

## Conflicts of interest

The authors declared no competing interests.

## Ethical considerations

Ethical issues (including plagiarism, misconduct, data fabrication, falsification, double publication or submission, redundancy) have been completely observed by the authors.

## Funding/Support

None.
